# Protocolized reduction of non-resuscitation fluids versus usual care in septic shock patients (REDUSE): a randomized multicentre feasibility trial

**DOI:** 10.1186/s13054-024-04952-w

**Published:** 2024-05-17

**Authors:** Anja Lindén, M. Spångfors, M. H. Olsen, J. Fisher, G. Lilja, F. Sjövall, M. Jungner, M. Lengquist, T. Kander, L. Samuelsson, J. Johansson, E. Palmnäs, J. Undén, J. Oras, M. Cronhjort, M. Chew, A. Linder, M. Lipcsey, N. Nielsen, J. C. Jakobsen, P. Bentzer, for the REDUSE Trial Group, Jane Fisher, Maria Nelderup, Lisa Hassel, Eva Johnsson, Camilla Claesson, Anna Lybeck, Susann Schrey, Linda K. Andersson, Sandra Holmström, Marina Larsson, Katarina Bramell, Karin Aspholm, Karin Olne, Hanna Larsson, Miklos Lipscey, Region Skåne, Niklas Nielsen

**Affiliations:** 1 Anesthesiology and Intensive Care, Department of Clinical Sciences Lund, Lund University, Lund, Sweden; 2 Department of Anesthesiology and Intensive Care, Helsingborg Hospital, Charlotte Yhléns Gata 10, 252 23 Helsingborg, Sweden; 3 Department of Anesthesiology and Intensive Care, Kristianstad Hospital, Kristianstad, Sweden; 4 Copenhagen Trial Unit, Centre for Clinical Intervention Research, The Capital Region, Copenhagen University Hospital - Rigshospitalet, Copenhagen, Denmark; 5 Department of Neuroanaesthesiology, The Neuroscience Centre, Copenhagen University Hospital -Rigshospitalet, Copenhagen, Denmark; 6 Neurology, Department of Clinical Sciences Lund, Lund University, Lund, Sweden; 7 Neurology Department, Skåne University Hospital, Lund, Sweden; 8 Department of Intensive and Perioperative Care, Skane University Hospital, Malmö, Sweden; 9 Department of Intensive and Perioperative Care, Skane University Hospital, Lund, Sweden; 10 Department of Anesthesiology and Intensive Care, Östersund Hospital, Östersund, Sweden; 11 Department of Operation and Intensive Care, Hallands Hospital Halmstad, Halmstad, Sweden; 12 Department of Anesthesiology and Intensive Care Medicine, Sahlgrenska Academy, University of Gothenburg, Gothenburg, Sweden; 13 Karolinska Institutet, Department of Clinical Sciences, Danderyd Hospital, Stockholm, Sweden; 14 Department of Anesthesiology and Intensive Care, Linköping University Hospital, Linköping, Sweden; 15 Infectious Diseases, Department of Clinical Sciences Lund, Lund University, Lund, Sweden; 16 Anaesthesiology and Intensive Care Medicine, Department of Surgical Sciences, Uppsala University, Uppsala, Sweden; 17 Hedenstierna Laboratory, Department of Surgical Sciences, Uppsala University, Uppsala, Sweden; 18 Department of Regional Health Research, The Faculty of Health Sciences, University of Southern Denmark, Odense, Denmark

**Keywords:** Septic shock, Fluid therapy, Non-resuscitation fluids, Randomized controlled trial, Feasibility

## Abstract

**Background/purpose:**

Non-resuscitation fluids constitute the majority of fluid administered for septic shock patients in the intensive care unit (ICU). This multicentre, randomized, feasibility trial was conducted to test the hypothesis that a restrictive protocol targeting non-resuscitation fluids reduces the overall volume administered compared with usual care.

**Methods:**

Adults with septic shock in six Swedish ICUs were randomized within 12 h of ICU admission to receive either protocolized reduction of non-resuscitation fluids or usual care. The primary outcome was the total volume of fluid administered within three days of inclusion.

**Results:**

Median (IQR) total volume of fluid in the first three days, was 6008 ml (interquartile range [IQR] 3960–8123) in the restrictive fluid group (n = 44), and 9765 ml (IQR 6804–12,401) in the control group (n = 48); corresponding to a Hodges–Lehmann median difference of 3560 ml [95% confidence interval 1614–5302]; *p* < 0.001). Outcome data on all-cause mortality, days alive and free of mechanical ventilation and acute kidney injury or ischemic events in the ICU within 90 days of inclusion were recorded in 98/98 (100%), 95/98 (98%) and 95/98 (98%) of participants respectively. Cognition and health-related quality of life at six months were recorded in 39/52 (75%) and 41/52 (79%) of surviving participants, respectively. Ninety out of 134 patients (67%) of eligible patients were randomized, and 15/98 (15%) of the participants experienced at least one protocol violation.

**Conclusion:**

Protocolized reduction of non-resuscitation fluids in patients with septic shock resulted in a large decrease in fluid administration compared with usual care. A trial using this design to test if reducing non-resuscitation fluids improves outcomes is feasible.

**Trial registration:**

Clinicaltrials.gov, NCT05249088, 18 February 2022. https://clinicaltrials.gov/ct2/show/NCT05249088

**Supplementary Information:**

The online version contains supplementary material available at 10.1186/s13054-024-04952-w.

## Introduction

Fluid is an essential component of care in patients with septic shock. Resuscitation fluids are administered to correct hemodynamic instability and ensure adequate tissue perfusion. All other fluids, such as vehicles for nutrition and medication, as well as fluids given to correct electrolyte disturbances and to ensure adequate hydration, may collectively be referred to as non-resuscitation fluids [1].

Several observational studies have reported that administering large volumes of fluid is associated with harmful effects [2–5]. This has stimulated numerous trials comparing protocolized reduction of resuscitation fluids with usual care in adult septic shock patients. These trials found no differences in clinical outcomes between groups and achieved relatively small differences in total fluid volumes [6]. However, as non-resuscitation fluids are the major source of fluid after the first day in the ICU, it stands to reason that targeting non-resuscitation fluids may more effectively reduce overall volume administration [1, 6, 7]. Modelling suggests that the volume of non-resuscitation fluids could be reduced by approximately three liters within the first five days after ICU admission [1]. This reduction is almost one liter greater than the maximum reduction achieved by protocols targeting resuscitation fluids [6]. The aim of this trial was to investigate if such a reduction is achievable in clinical practice, as it may potentially impact patient-centered outcomes. Accordingly, we conducted the protocolized REDUction of non-resuscitation fluid versus usual care in SEptic shock patients (REDUSE) Feasibility Trial to assess fluid volume administration, outcome reporting, and protocol adherence of a protocolized reduction in administration of non-resuscitation fluids compared with usual care in patients with septic shock.

## Methods

### Trial design

The REDUSE Feasibility Trial was an investigator-initiated, multicenter, parallel-group randomized trial. The trial protocol and the statistical analysis plan have been published previously [8, 9]. All procedures followed the ethical standards of the 1964 Helsinki declaration and its later amendments. The trial protocol was approved by the Swedish ethics review authority (#2020-06594, 08 August 2021) and registered at clinicaltrials.gov (NCT05249088, 18 February 2022) prior to trial initiation. We primarily used a deferred consent process and obtained informed consent from all surviving participants. All authors vouch for the completeness of the data and the fidelity of the trial to the protocol.

### Participants

Adult patients (≥ 18 years of age) with septic shock (suspected/confirmed infection, plasma lactate > 2 mmol/l and infusion of vasopressor to maintain MAP > 65 mmHg after adequate fluid resuscitation) within 12 h of admission to the ICU and ongoing vasopressor therapy at the time of inclusion were eligible for inclusion [10]. Initially, we also mandated that patients should have fulfilled criteria for septic shock within the three hours preceding inclusion. To promote patient inclusion, the latter criterion was dropped after the inclusion of seven patients. The exclusion criterion was suspected or confirmed pregnancy.

### Randomization

Eligible patients were identified by the clinician caring for the patient and were randomized using a centralized, internet-based computer-generated allocation tool, stratified by site. Allocation was performed in a 1:1 ratio in permuted blocks of varying size to either protocolized restriction of non-resuscitation fluids or usual care. The clinical team caring for the patient was not blinded to the assigned intervention due to the nature of the intervention. Study participants, their relatives, trial statisticians, and outcome assessors at 90-days and six months were blinded to the assigned intervention.

### Intervention

In the restrictive fluid group, maintenance fluid was discontinued in participants who were in a positive cumulative balance and judged not to be dehydrated by the treating physician. The time interval for assessment of fluid balance was left at the discretion of the treating physician. Participants deemed to have a neutral or negative cumulative balance received fluid to cover their daily need for water (1 ml/kg/h) and ongoing losses. Enteral nutrition was administered according to local practice but always at an energy density of at least 2 kcal/ml. Glucose could be used at a maximum dose of 1 g/kg/day and a concentration of 20% or greater as nutrition, starting at the earliest at 72 h from inclusion, if enteral nutrition was not tolerated. In participants with insulin-dependent diabetes mellitus, glucose solutions could be started earlier if enteral feeding was not tolerated and if mandated by local protocol. Parenteral nutrition was administered according to local routines, and enteral water/intravenous fluid could be given as needed to correct electrolyte imbalances. Intravenous medications were concentrated according to a trial-specific protocol developed by the investigators in collaboration with two pharmacists and was based on the most concentrated solutions found in the literature (please see Supplementary Material, Appendix A). Volume used to flush lines after injections were administered according to local practice.

Participants pragmatically received non-resuscitation fluids in the usual care group according to local practice. Unless local protocols stated otherwise, maintenance fluid (crystalloids and/or glucose solutions and/or enteral water) was given at a dose of 1 ml/kg/h. Glucose solutions were used at a maximum concentration of 10%, medications, enteral and parenteral nutrition was administered according to local guidelines.

In both groups, resuscitation fluids were administered according to the Surviving Sepsis Campaign (SSC) guidelines in the salvage and optimization phase and according to local protocol in the stabilization and de-escalation phases [11, 12]. In participants who required surgery, fluids were administered at the discretion of the anesthetist. The assigned interventions continued for the duration of the ICU stay, up to a maximum of 90 days.

### Outcomes

#### Feasibility outcomes

The primary feasibility outcome was total volume of fluid administered within three days of randomization (D0–D3). The secondary feasibility outcomes were (a) proportion of participants with outcome data on all-cause mortality, days alive and free of mechanical ventilation, acute kidney injury and ischemic events in the ICU (cerebral, cardiac, intestinal or limb ischemia) within 90 days of inclusion (please see Supplementary Material for definitions), (b) proportion of surviving participants assessed for health-related quality of life (HRQoL) by the EQ-5D five level version (EQ-5D-5L) and the Montreal Cognitive Assessment (MoCA) at six months after inclusion, (c) proportion of eligible patients who were randomized and consented, (d) proportion of participants experiencing at least one protocol deviation [13–16].

#### Exploratory clinical outcomes

Primary exploratory clinical outcomes were all-cause mortality at 90 days from inclusion, ≥ 1 complication in the ICU (acute kidney injury or ischemic events), days alive and free of mechanical ventilation within 90 days of inclusion, cognitive function measured by MoCA, and HRQoL measured by EQ-5D-5L visual analog scale (VAS) at six months. Secondary exploratory clinical outcomes include volumes of resuscitation and non-resuscitation fluids and cumulative fluid balance at day three and five after inclusion, hemodynamic stability (highest lactate, highest dose of noradrenaline and highest cardiovascular SOFA-score) and use of diuretics during the first five days from inclusion, renal function within 90 days from inclusion and functional outcome as measured by the Glasgow Outcome Scale Extended (GOSE) at six months from inclusion [17]]. In addition, we registered incidence of hypoglycemia (≤ 3.9 mmol/l), electrolyte/metabolic disturbances and suspected unexpected serious adverse complications in the ICU (see Supplementary Material for details).

#### Sample size

Our previous study suggested that the total volume of fluid may be reduced by a median of 3.1 (interquartile range [IQR] 1.5–5.0 and standard deviation of 2.8) liters in the first 3 days after ICU admission by restrictive administration of non-resuscitation fluids [1]. Based on the standard deviation derived above we needed 42 participants in each group for the trial to have a power of 90% at an alpha level of 0.05 to detect a difference of 2 L between the groups. To account for data not being normally distributed, we included 15% more participants than the calculated sample size, resulting in a total sample size of 98 participants in the trial [18].

Thresholds for the secondary feasibility outcomes were: proportion of participants with clinical outcome data on all-cause mortality, days alive and free of mechanical ventilation and acute kidney injury and ischemic events in the ICU: 95% (95% confidence interval [CI] 89–98); proportion of surviving participants assessed by the EQ-5D-5L and MoCA at six months after inclusion: 85% (95% CI 73–92); proportion of eligible patients who were randomized and consented: 75% (95% CI 67–81); and proportion of participants experiencing at least one protocol deviation: 10% (95% CI 6–18).

### Statistical analysis

Two statisticians performed all analyses independently according to the statistical analysis plan before unblinding data. The analyses were performed according to an intention-to-treat principle and adjusted for participating site [9]. The primary feasibility outcome was analyzed using the van Elteren test and is presented with median differences with 95% Hodges–Lehman confidence intervals (95% HLCI). All available data was used in the primary analysis. The secondary feasibility outcomes are presented as percentages with confidence intervals calculated using the 1-sample proportions test without continuity correction. For the exploratory clinical outcomes, we analyzed count data outcomes using the van Elteren test with adjustment for site, continuous outcomes using mixed effects linear regression with site as random intercept, and dichotomous outcomes using mixed effect logistic regression with site as random intercept and relative risks obtained using the ‘nlcom’ Stata command and/or by G-computation in R. Due to the exploratory nature of the trial, *p* values were not adjusted for multiple comparisons. All statistical analyses were performed using Stata v. 17 (StataCorp LLC, Texas, USA) and/or R v. 4.2.1 (R Core Team, Vienna Austria). A *p* value of 0.05 or less is considered statistically significant.

## Results

### Patient characteristics

**Fig. 1:**
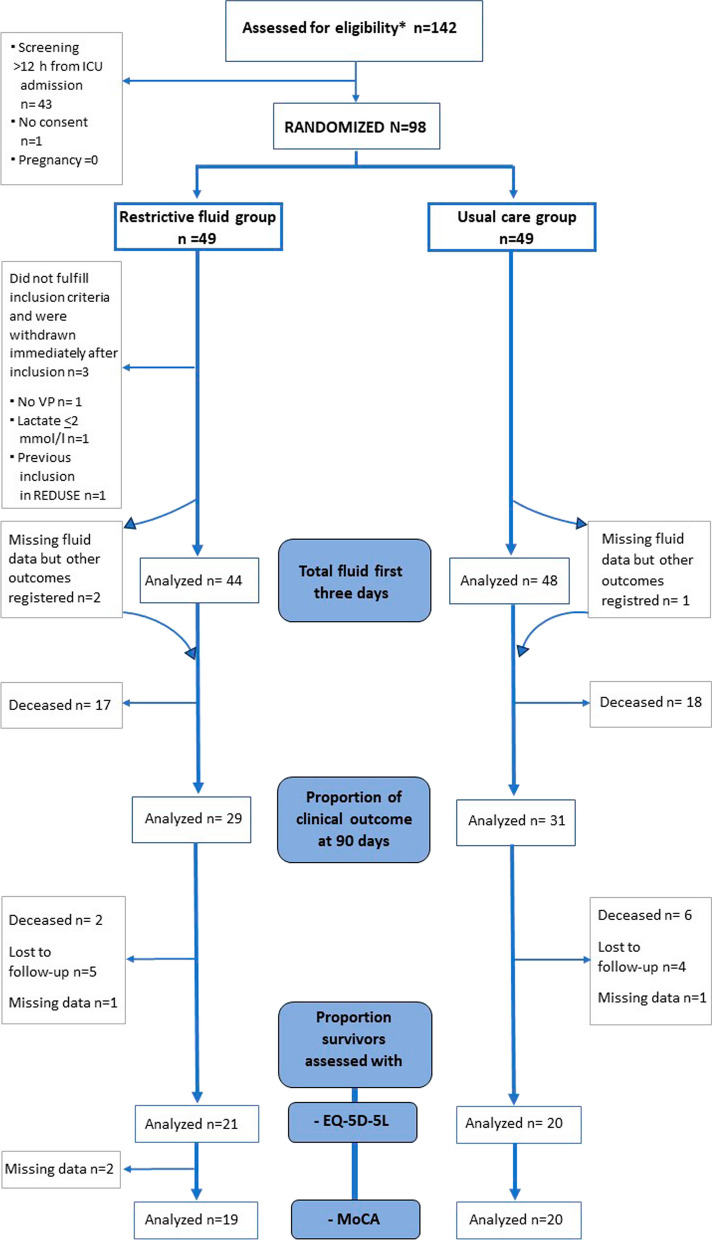
CONSORT flowchart. ^*^Includes eight patients that were not eligible. Patients are labelled “lost to follow-up” if *all* outcome data were missing. If *some* of the outcome data were missing, the patient is labelled as “missing data”. VP: vasopressor

Between 7 March and 13 September 2022, eligibility was assessed in 142 adult patients admitted with septic shock in six Swedish ICUs. In 43 patients, the 12-h inclusion window had passed at the time of screening and one patient declined participation in the trial. A total of 49 patients were randomized to receive protocolized reduction of non-resuscitation fluids, and 49 patients were randomized to usual care. Immediately after randomization, three participants were identified as not fulfilling the inclusion criteria, and they were withdrawn from the trial. Three participants had incomplete fluid charts. Consequently, data on the primary outcome were available from 44 participants (94%) in the restrictive fluid group and 48 participants (98%) from the usual care group, all of whom received the allocated treatment (Fig. 1). The last follow-up was performed on 24 April 2023. Baseline characteristics are presented in Table 1**.**

**Table 1:** Baseline characteristics

	Restrictive fluid group (n = 46)	Usual care (n = 49)	All participants (n = 95)
Age, yrs	75 (59–80)	68 (60–78)	72 (59–79)
Female sex	20 (43)	19 (39)	39 (41)
Height, cm	171 (163–181)	170 (167–178)	171 (164–180)
Weight, kg	81 (70–97)	79 (64–93)	80 (69–95)
Charlson comorbidity index	2 (0–3)	2 (1–3)	2 (1–3)
Time from ICU admission to inclusion, hrs	3.7 (1.4–8.3)	3.4(1.2–7.8)	3.5 (1.2–7.8)
Frailty score	4 (3–6)	4 (3–6)	4 (3–6)
SAPS III	72 (63–85)	71 (62–83)	72 (62–83)
SOFA-score	10 (8–13)	9 (7–12)	10 (7–12)
Source of ICU admission			
ED Hospital ward OR Other ICU	16 (35) 18 (39) 11 (24) 1 (2)	19 (39) 23 (47) 7 (14) 0	35 (37) 41 (43) 18 (19) 1 (1)
Site of infection			
Pulmonary Urinary tract Abdominal Gastrointestinal Skin/soft tissue Bone and joint CNS	13 (28) 12 (26) 19 (41) 4 (9) 5 (11) 0 0	17 (35) 7 (14) 16 (33) 5 (10) 3 (6) 2 (4) 0	30 (32) 19 (20) 35 (37) 9 (9) 8 (8) 2 (2) 0
Lactate, mmol/l	3.8 (2.4–6.5)	3.4 (2.5–6.1))	3.7 (2.4–6.1)
Creatinine, μmol/l	165 (110–242)	150 (86–280)	155 (99–260)
Dose of noradrenaline at inclusion, mcg/kg/min	0.22 (0.13–0.43)	0.26 (0.14–0.40)	0.23 (0.14–0.40)
Volume of IV fluids 24 h before inclusion	3840 (2183–5013)	3550 (3000–5117)	3825 (2500–5025)
Use of respiratory support at inclusion	24 (52)	21 (43)	45 (47)
Surgery prior to ICU admission	14 (30)	14 (29)	28 (29)
Length of first ICU day (D0), hours	12 (7–19)	9 (5–17)	10 (5–17)

Data presented as median (interquartile range) or no (%) as appropriate. SAPS: Simplified Acute Physiology Score III, SOFA: Sequential Organ Failure Assessment at ICU admission (worst value ± 1 h of ICU admission), ED: emergency department, OR: Operating Room, CNS: Central Nervous System. Site of infection: Please note that participants can have multiple sites of infection. Respiratory support: Non-invasive or invasive mechanical ventilation. Laboratory values: the value closest in time to enrolment, within ± 6 h, was registered

### Primary feasibility outcome

**Fig. 2:**
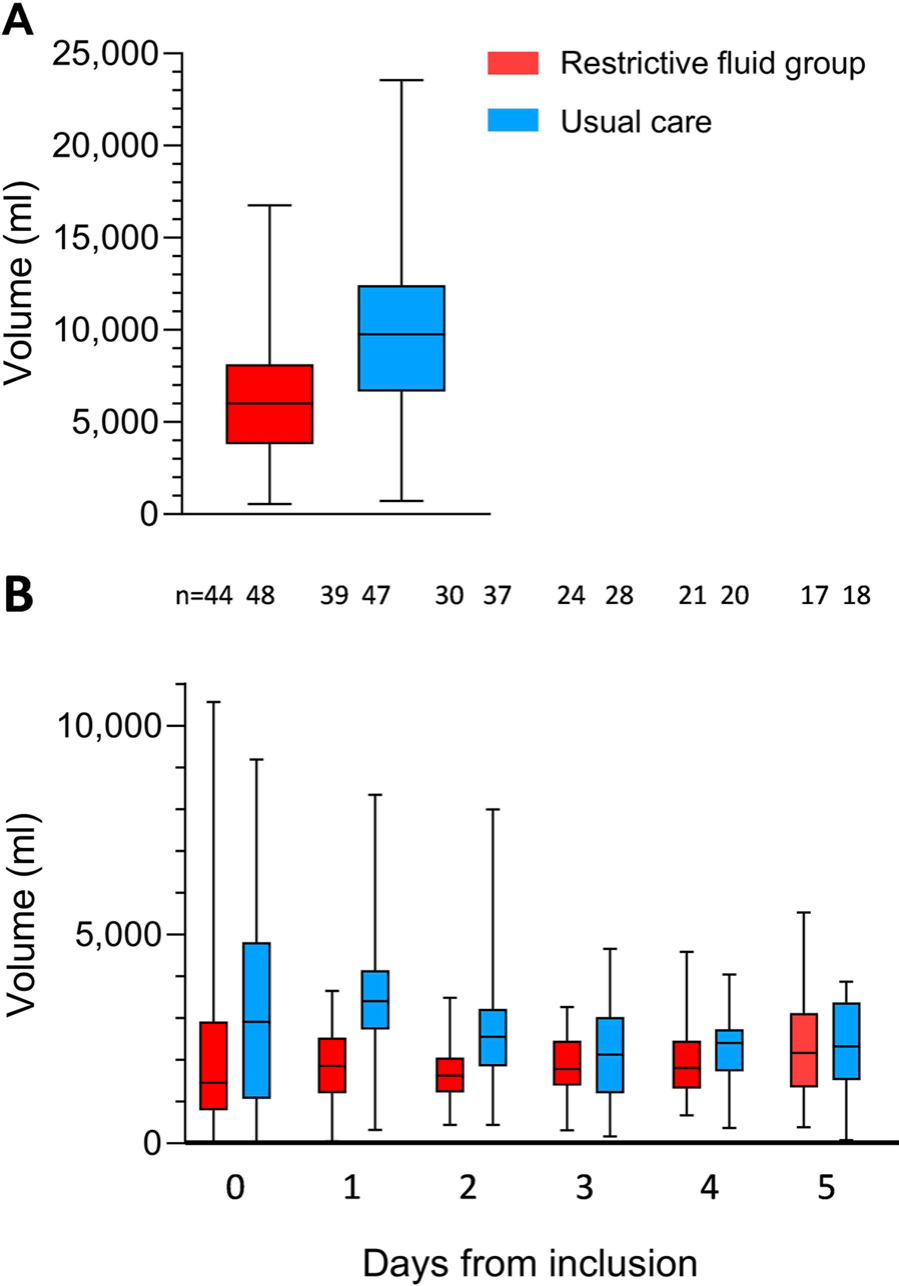
**A**: Total volume of fluids administered during the first three days (D0-3) in the ICU. **B**: Total daily fluid volume the first five days in the ICU. n indicates number of patients

The median length of stay in the ICU was 67 (IQR 32–169) hours in the restrictive fluid group and 72 (IQR 40–145) hours in the usual care group. Within the first three days of inclusion, participants in the restrictive fluid group received a median of 6008 (IQR 3960–8123) ml of total fluids and the usual care group received 9765 (IQR 6804–12,401) ml. The Hodges–Lehman median difference between groups was − 3560 (95% HLCI − 5302 to − 1614, *p* < 0.001) ml (Fig. 2A and B). The electronic supplementary material presents details regarding the volume and composition of non-resuscitation fluids in the restrictive and usual care groups (Supplementary Material, Fig S1 and Table S1). A total of 40 patients had either been discharged (n = 30) or died (n = 10) before day 3. In a sensitivity analysis of participants admitted to the ICU for three days or more (complete case analysis), participants in the restrictive fluid group (n = 24) received 7144 (IQR 5872–8582) ml and participants in the usual care group (n = 28) received 12,237 (IQR 9932–14,268) ml in the first three days—a median difference of − 4749 (95% HLCI − 6507 to − 2879, *p* < 0.001) ml. See Supplementary Material, Table S2–3 for additional sensitivity analyses regarding daily/hourly fluid data and for data on total volume of fluids within the first 4 and 5 days of inclusion.

### Secondary feasibility outcomes

The proportions of participants with outcome data on all-cause mortality, days alive and free of mechanical ventilation, any acute kidney injury or ischaemic events in the ICU within 90 days of inclusion were 98/98 (100%), 95/98 (98%), and 95/98 (98%), respectively. The proportion of randomized patients who survived to six months and were assessed by MoCA was 39/52 (75%) and by EQ-5D-5L was 41/52 (79%). The proportion of all eligible patients who were randomized was 90/134 (67%), and the proportion of randomized participants experiencing at least one protocol deviation was 15/98 (15%) (Table 2 and Supplementary Material, Table S4). The protocol deviations were inclusion of a non-eligible patient (n = 8) and treatment temporarily not given according to protocol (n = 7).

**Table 2:** Secondary feasibility outcomes

Proportion of eligible patients who were randomized and consented	67 (59–75)
Within 90 days from inclusion	
Proportion of randomized participants experiencing at least one protocol deviation	15 (10–24)
Proportion of participants with clinical data on	
All-cause mortality	100 (96–100)
Days alive and free of mechanical ventilation	98 (93–99)
Acute kidney injury and ischemic events	98 (93–99)
At 6 months from inclusion	
Proportion of participants with clinical data on	
MoCA	75 (62–85)
EQ-5D-5L VAS	79 (66–88)

Data presented as percent (95%CI)

MoCA: Montreal Cognitive Assessment, EQ-5D-5L: European Quality of life 5-Dimensions 5-Levels visual analogue scale

### Exploratory clinical outcomes and harms

All-cause mortality at 90 days, days alive and free of mechanical ventilation, AKI and ischemic complications, cognitive function and HRQoL did not differ between the groups (Table 3). The total volume of non-resuscitation fluids on days 3 and 5 was lower in the restrictive fluid group than in the usual care group (median difference − 3316 [95% HLCI − 4833 to 1954, *p* < 0.001] and − 3191 [95% HLCI − 5199 to − 1166, *p* = 0.006] ml, respectively), whereas the total volume of resuscitation fluids on days 3 and 5 did not differ between groups (0 [95% HLCI − 750 to 627, *p* = 0.77] and 37 [95% CI − 742 to 680, *p* = 0.96] ml, respectively). Cumulative fluid balance on day 3 and 5 was lower in the restrictive fluid group than in the usual care group (− 2109 [95% HLCI − 3480 to − 831, *p* < 0.001] and − 1812 [− 3140 to − 502] ml, respectively) (Table 3; Supplementary Material, Table S5 and Fig S3). Hypoglycemia occurred in 10 participants in the restrictive fluid group and in four participants in the usual care group. One episode in the restrictive fluid group was severe (blood glucose ≤ 2.2 mmol/l). Hyperchloremic acidosis developed in one participant in the restrictive fluid group and one in the usual care group, hypernatremia and metabolic alkalosis in one participant in the restrictive fluid group and none in usual care group. No suspected unexpected severe adverse complications (SUSAC) occurred in either group (Supplementary Material, Table S6).

**Table 3:** Primary exploratory clinical outcome

	Restrictive fluid group	Usual care	Relative risk (95% CI)	*p*
At 90 days from inclusion				
All-cause mortality	17/46 (37)	18/49 (37)	1.01 (0.79–1.23)	0.97
One or more complications (AKI or ischemic event)	39/46 (85)	38/49 (78)	0.97 (0.95–1.07)	0.37
Days alive and free of mechanical ventilation	86 (3–90)	86 (9.5–90)	–	0.97
At 6 months from inclusion				
MoCA	19 (17–21)	19 (18–20)	–	0.72
EQ-5D-5L VAS	70 (50–80)	70 (58–81)	–	0.89

Data presented as median (interquartile range) or fraction (%)

AKI: Acute Kidney Injury, MoCA: Montreal Cognitive Assessment, EQ-5D-5L VAS: European Quality of life 5-Dimensions 5-Levels visual analogue scale

## Discussion

In this multicenter feasibility trial in patients with septic shock in the ICU, the protocolized reduction of non-resuscitation fluids reduced cumulative administration by approximately 3.6 L in the first 3 days compared with usual care. The secondary feasibility thresholds were reached for the proportion of participants with clinical data at 90 days. In contrast, the thresholds were not reached for the proportion of survivors assessed for cognitive function and HRQoL at six months, the proportion of participants with protocol deviations and the proportion of eligible patients who were randomized and consented.

The intervention reduced fluid administration by approximately 3.6 L, almost twice as large as the reduction observed in the most fluid-restrictive trial limiting resuscitation fluids in ICU patients with septic shock [19]. The reduction was even larger in participants staying in the ICU for three days or more, just below 5 L. This result supports our hypothesis that fluid administration in septic shock can be effectively reduced by targeting non-resuscitation fluids. Naturally, the separation between the groups is influenced not only by the intervention but also by the fluid volumes administered in the usual care group, potentially limiting the external validity of our findings. Clinical practice for administering non-resuscitation fluids is often not protocolized and differs from unit to unit, therefore, this caveat is particularly relevant [1]. In the CLASSIC trial, which investigated the effects of reducing the administration of resuscitation fluids on patient-centered outcomes in septic shock, participants in the control group received a total volume of 10.8 L during days 1–5 in the ICU [19]. Similarly, total volume of fluids in our previous observational study in Canadian and Swedish ICUs was 10.8 L during days 1–5 in the ICU [1]. These previous data are consistent with the finding that the usual care group in the present trial received 10.6 L in the corresponding time period, suggesting that fluid administration in this usual care group is representative of a larger population of critically ill patients (Supplementary Material, Table S3). Thus, excessive fluid administration in the usual care group in our trial is unlikely and cannot explain the observed separation between the groups.

No guidelines specify follow-up rates regarding cognitive function and HRQoL in ICU survivors. Most ICU trials have reported follow-up rates of 70–80%, although higher rates up to 91% have been reported in recent years [20–23]. Our feasibility thresholds for these outcomes were set at the higher end of previously reported rates. We believe the chosen thresholds represent a reasonable compromise between what is achievable and what is needed for the results to be valid. Not reaching these thresholds could introduce selection bias, and we conclude that more extensive efforts should be made to minimize missing cognitive function and HRQoL data in a future trial. Such efforts could include using more regular central data monitoring to allow timely identification of sites with the need of more training and support [24].

We did not meet the feasibility thresholds for protocol deviations or the proportion of eligible patients who were randomized and consented. Not reaching these predefined thresholds may partly be explained by the proportionally long run-in time, an inherent feature of a small trial conducted at multiple sites. In a longer-running trial, increased familiarity with the trial will lessen the impact of the run-in period. Advanced training and further development of the inclusion process represent additional opportunities for improving the inclusion rate and protocol adherence.

The intervention reduced the administration of glucose solutions, which could have exposed the participants to an increased risk of hypoglycemia. In several countries, Sweden included, glucose solutions are routinely administered to critically ill patients to prevent hypoglycemia and ketosis, but practices vary between different countries and this prevention strategy is not mentioned in nutritional guidelines for critically ill patients [1, 25–29]. Our intervention could, in theory, also result in a lower intravascular fluid volume, which could precipitate ischemic events and acute kidney injury. In addition, the use of concentrated drugs could expose patients to an increased risk of drug stagnation and obstruction of central venous catheters if lines are not appropriately flushed. None of the potential adverse effects mentioned above were detected, but clinically important adverse effects of the intervention cannot be excluded due to the limited sample size.

In this feasibility trial, we did not detect any effects on patient-important outcomes; however, the present intervention resulted in a larger reduction in fluid administration than in previous trials, particularly in patients with a longer ICU stay. Moreover, the type and number of adverse effects due to our intervention may differ from those seen in trials primarily focused on reducing the administration of resuscitation fluids [19]. Based on this we believe that a trial powered to detect effects on patient-important outcomes and potential adverse effects is warranted.

### Strengths and limitations

The strengths of our trial include the randomized, multicenter trial design and the fact that a prespecified protocol and statistical analysis plan were followed. Moreover, having few exclusion criteria increases the generalizability of the trial. A limitation of the trial is our pragmatic definition of usual care meaning that the administration of non-resuscitation fluids in the control group was not strictly protocolized and may have varied from site to site. This approach was taken to yield trial results that would be pertinent to the current care practices in the ICU for patients with septic shock and because protocolizing usual care is not a straightforward alternative, as no guidelines or definitions describe what constitutes usual care for administering non-resuscitation fluids. However, we acknowledge that the pragmatic definition of usual care may limit external validity of our results. Also, as mentioned in the methods section, inclusion criteria were modified slightly after the inclusion of seven patients. Theoretically, the change could have entailed that patients with a lower lactate at the time of randomization were included and we cannot exclude that this may have influenced the patient population. However, given that change in inclusion criteria was implemented early in the trial, such an influence is likely to be minor. In addition, we did not know cognitive function and health-related quality of life at baseline and we cannot exclude that baseline differences in these outcomes may have influenced our results. However, such data are typically not available in trials on critically ill patients and randomization should provide reasonably balanced groups also with regard to these parameters. All sites routinely flushed lines after drug injection but we did not protocolize flushing volumes. Theoretically, low flushing volumes could have resulted in underdosing of the concentrated drugs, such as antibiotics, in the intervention group. Lastly, another potential limitation is that the staff who treated the participants were not blinded to the allocated treatment due to the nature of the intervention.

## Conclusion

In patients with septic shock, protocolized reduction of non-resuscitation fluids resulted in a large decrease in fluid administration compared with usual care. A trial using this design to test the hypothesis that reducing non-resuscitation fluids improves patient-important outcomes seems feasible.

### Supplementary Information

**Additional file 1**. (PDF 774 kb)

## Data Availability

Individual de-identified data is available for sharing with researchers who provide a methodologically sound proposal to the corresponding author. To gain access, data requestors will need to sign a data access agreement.
