# Supplementary material for: Protocolized reduction of non-resuscitation fluids versus usual care in septic shock patients (REDUSE): a randomized multicentre feasibility trial
Source: Crit Care. 2024 May 17;28:166. doi: 10.1186/s13054-024-04952-w (PMC11100208; doi:10.1186/s13054-024-04952-w)
Supplement: Supplementary file 1 — Additional file 1. (PDF 774 kb) [file 13054_2024_4952_MOESM1_ESM.pdf]

Supplementary Material

This appendix has been approved by the authors to give readers additional information about their work.

Supplement to: Lindén A, Spångfors M, Olsen MH et al. Protocolised REDUction of non-resuscitation fluids versus usual care in SEptic shock patients (REDUSE): a randomized multicentre feasibility trial

TABLE OF CONTENTS

TRIAL PERSONNEL 4

Investigators 4

Trial organizations and management 4

SUPPLEMENTARY METHODS 4

Investigator responsibilities 4

Data collection and verification 5

Details of the intervention 5

Differences between the statistical report and the presented results 5

Definitions 5

Fluids 5

Complications 5

Protocol deviations 6

Frailty score 6

Site of infection 6

Statement on data sharing 6

SUPPLEMENTARY RESULTS 7

TABLES 7

TABLE S1. VOLUME OF NON-RESUSCITATION FLUIDS ADMINISTERED THE FIRST THREE DAYS (D0-D3), ACCORDING TO TREATMENT GROUP. 7

TABLE S2. SENSITIVITY ANALYSES OF THE PRIMARY OUTCOME 7

TABLE S3. CUMULATIVE TOTAL VOLUME ADMINISTRATION – ALL AVAILABLE DATA 8

TABLE S4. PROTOCOL DEVIATIONS. 8

TABLE S5. SECONDARY EXPLORATORY CLINICAL OUTCOME. 8

TABLE S6. COMPLICATIONS AND HARMS IN THE ICU UP TO 90 DAYS AFTER INCLUSION 9

TABLE S7. POST HOC-ANALYSES OF EXPLORATORY OUTCOMES. 10

TABLE S8. ACUTE KIDNEY INJURY STAGE WITHIN 90 DAYS 11

TABLE S9. PARTICIPATING HOSPITALS AND NUMBER OF RANDOMIZED PATIENTS. 11

FIGURES 12
FIGURE S1. DAILY NON-RESUSCITATION FLUIDS. 12
FIGURE S2. DAILY RESUSCITATION FLUIDS. 12
FIGURE S3. CUMULATIVE FLUID BALANCE. 13
FIGURE S4. CUMULATIVE TOTAL FLUID ADMINISTRATION 13
FIGURE S5. SURVIVAL 90 DAYS FROM INCLUSION. 14
APPENDIX A 15
DILUTIONS OF MEDICATIONS IN THE INTERVENTION GROUP. 15
APPENDIX B 24
TREATMENT ALGORITHM FOR NON-RESUSCITATION FLUIDS IN THE INTERVENTION GROUP. 24
REFERENCES 27

TRIAL PERSONNEL

Investigators

Helsingborg Hospital: Peter Bentzer (Principal Investigator); Anja Lindén (PSI), Jane Fischer (Trial coordinator), Niklas Nielsen, Maria Nelderup (Coordinator of patient reported outcomes for all sites), Lisa Hassel. *Kristianstad Hospital:* Martin Spångfors (PSI), Eva Johnsson, Camilla Claesson. *Skane University Hospital, Lund:* Thomas Kander (PSI), Gisela Lilja (Senior coordinator of patient reported outcomes), Maria Lengquist, Adam Linder, Anna Lybeck, Susann Schrey, Linda K Andersson. *Skane University Hospital, Malmö:*
Fredrik Sjövall (PSI), Mårten Jungner, Sandra Holmström, Marina Larsson, Katarina Bramell. *Östersund Hospital*: Line Samuelsson (PSI), Joakim Johansson, Karin Aspholm *Hallands Hospital Halmstad*: Johan Undén (PSI), Eva Palmnäs, Karin Olne, Hanna Larsson. *Danderyd Hospital, Stockholm:* Maria Cronhjort. *Uppsala University Hospital:* Miklos Lipscey. *Linköping University Hospital:* Michelle Chew. *Sahlgrenska University Hospital*: Jonatan Oras. *Copenhagen Trial Unit*: Janus Christian Jakobsen (Chief Statistician), Markus Harboe Olsen.
PSI – Principal Site Investigator

Trial organizations and management

Trial organizations:
Region Skåne, Helsingborg Hospital, Helsingborg, Sweden (Sponsor)
Lund University, Lund, Sweden

Management group:
Peter Bentzer, Niklas Nielsen, Janus Christian Jakobsen, Gisela Lilja, Jane Fisher, Anja Lindén.

Steering group:
Management group and principal site investigators as indicated above.

Statisticians:
Janus Christian Jakobsen (Chief statistician), Markus Harboe Olsen

SUPPLEMENTARY METHODS

Investigator responsibilities

The trial steering group designed the trial. Principal site investigators vouch for the data recorded at each hospital. Data analysis was independently performed by two statisticians (Janus C Jakobsen and Markus H Olsen). A final statistical report was written after consensus was achieved. The steering group vouches for the accuracy and completeness of the data and analysis and for the adherence of this report to the trial protocol and the statistical analysis plan.
The initial version of the manuscript was drafted by the first and last authors and was further developed and approved by all authors. The funders had no role in the analysis of the data, in the preparation or approval of the manuscript, or in the decision to submit the manuscript for publication.

Data collection and verification

Data for the baseline characteristics and primary and secondary outcome measures were obtained from hospital charts, except for the six-month follow up. Assessment of functional outcome at the six-month follow-up was made at a face-to-face follow-up or by telephone contact with patients or their relatives.
The trial was externally monitored by national monitoring offices coordinated by the clinical trial manager and Clinical Studies Sweden, Forum South. All variables were collected in a patient-specific trial ledger or directly in an electronic case report form (eCRF) which was created in collaboration with Spiral Software (New Zealand). Site principal investigators were responsible for training of clinical staff on how to enter variables correctly. Special emphasis was placed on how to record fluid administration and fluid balance in a standardized manner. Instructions were available in the trial ledger and in the eCRF. All sites had a digital site initiation meeting with monitors before start of inclusion and an on-site meeting at end of study. The meetings included control of routines for data collection and data entry as well as quality control of data by comparing selected source data with data entered in eCRF. The principal site investigator was responsible for ensuring that all relevant data were entered into the eCRF. To promote data quality, the eCRF had several inbuilt mechanisms to prevent data entry errors such as range checks for data values.

Details of the intervention

Patients received non-resuscitation fluids according to their allocated treatment arm as soon as possible, at the latest within two hours of inclusion. Prior to initiation of the trial, site investigators established what constituted 'usual care' at their site, to limit drift in the usual care group.

Differences between the statistical report and the presented results

In the statistical report, the analysis of protocol deviations including the three participants that were randomized without fulfilling the inclusion criteria and immediately withdrawn was erroneously classified as a sensitivity analysis. This analysis should have been classified as the primary analysis and is presented as such.

Definitions

Fluids

Fluid balance was calculated as sum of all input of enteral and parenteral fluids minus all measured losses. Estimated loss through evaporation was not included in fluid balance. Stool was not included in the fluid balance unless the patient had a faecal management system or similar device in place. Crystalloids were classified as resuscitation fluids if administered to correct hemodynamic impairment as noted in the patient chart or given at a rate > 5 ml/kg/h (Finfer 2010).

Complications

Complications were defined as follows:

- acute kidney injury, according to the KDIGO criteria (Kellum-13). In patients without data on creatinine prior to hospital admission, the baseline creatinine was estimated using the chronic kidney disease epidemiology (CKD-EPI) equation (Inker -21).

- ischemic events in the ICU (cerebral, cardiac, intestinal or limb ischemia) within 90 days of inclusion. Cerebral ischemia was defined as ischemia seen on magnetic resonance imaging (MRI) or computer tomography (CT) scan; cardiac ischemia as myocardial infarction/unstable angina AND treatment as a consequence – percutaneous coronary intervention (PCI)/thrombolysis or initiation/increased antithrombotic treatment; intestinal ischemia as diagnosed during surgery or by angiography; limb ischemia if in combination with treatment - open/percutaneous vascular intervention, amputation, initiation of/increased antithrombotic treatment

In addition to the patient-centred complications above, the following complications were registered:

- Hypoglycaemia (≤ 3.9 mmol/l)
- Electrolyte and metabolic disturbances (hypernatremia > 159 mmol/L, hyperchloremic acidosis [pH < 7.15 and plasma Cl^- > 115], metabolic alkalosis [pH > 7.59 and standard base excess (S-BE) > 9])
- Suspected unexpected serious adverse complication (SUSAC) - an adverse event not reasonably explained by factors other than the intervention which may cause death, or be life threatening, prolong hospitalisation, or may result in significant disability/incapacity.

Protocol deviations

Protocol deviations include randomization of a non-eligible patient and non-compliance with the treatment algorithm in the intervention arm.

Frailty score

Frailty score refers to the first version, described by Rockwood et al (Rockwood -05).

Site of infection

The site of infection was characterized according to the Mellhammar-Linder criteria (Mellhammar -22).

Statement on data sharing

Beginning nine months after publication of the main report of this trial individual de-identified data will be available for sharing with researchers who provide a methodologically sound proposal as judged by the steering committee. To gain access, data requestors will need to sign a data access agreement. Proposals should be directed to the principal investigator via email: peter.bentzer@med.lu.se and will be reviewed by the REDUSE-trial steering group.

SUPPLEMENTARY RESULTS

SENSITIVITY ANALYSES

The generalized estimating equation resulted in a difference of 945 (95% CI 556-1335 p<0.001) ml between the restrictive fluid group and usual care group. The results of the other sensitivity analyses are shown below, in **Table S2**.

TABLES

Table S1. Volume of non-resuscitation fluids administered the first three days(D0-D3), according to treatment group.

Non-resuscitation fluids	Restrictive fluid group (n= 44)	Usual care (n=48)
Vehicle, ml	1833 (748-2521)	2588 (1286-3726)
Crystalloid, ml	0 (0-145)	20 (0-1000)
Glucose, ml	117 (0-606)	2605 (1634-3693)
Parenteral, ml	0 (0-0)	0 (0-0)
Enteral nutrition, ml	146 (0-919)	200 (0-659)
Enteral water, ml	310 (0-1095)	135 (0-796)
Enteral water for correcting electrolyte disturbances, ml	0 (0-0)	0 (0-0)

Data presented as median (interquartile range).

Table S2. Sensitivity analyses of the primary outcome

	Restrictive fluid group	Usual care	Median difference (95%HLCI)	p
Average daily, ml	1787 (1468-2187) *n=44*	2750 (2011-3497) *n=48*	-883 (-1300 to -425)	<0.001
Average hourly, ml	95 (72-134) *n=44*	155 (113-180) *n=48*	-48 (-69 to -26)	<0.001
Complete case analysis, ml	7144 (5872-8582) *n=24*	12237 (9932-14268) *n=28*	-4749 (-6507 to -2879)	<0.001
Per-protocol, ml	6008 (3704-8123) *n=40*	9645 (6734-12323) *n=47*	-3560 (-5395 to -1549)	<0.001

Total fluid administered in the first three days (D0-D3) presented in various time frames. Data for the restrictive fluid group and usual care are presented as the median (interquartile range). Differences are presented as median differences with 95% Hodge Lehmann (HL) confidence intervals (CI). Complete case analysis: included patients still admitted on the third ICU day (D0-3). Per-protocol: including only patients whom fulfilled the inclusion criteria and received the intervention.

Table S3. Cumulative total volume administration – all available data

	Restrictivefluid group (n=44)	Usual care (n=48)
D0-D4, ml*	7206 (4119-9793)	10579 (6723-14684)
D0-D5, ml	7599 (4110-12162	11000 (6723-16920)

Data presented as median (interquartile range). D: Day. * corresponds to Day 1-5 in the CLASSIC trial and in our previous observational trial (Lindén-Søndersø et al. 2019).

Table S4. Protocol deviations.

Protocol deviation	Proportion of patients with at least one such deviation
Did not receive the allocated treatment	0 (0)
Not eligible	8 (8)
- Age <18 yrs	0 (0)
- Randomized >12 hrs after ICU admission	5 (5)
- Pregnant	0 (0)
- No septic shock	2 (2)
- Previously included in REDUSE	1 (1)
Treatment temporarily not given according to protocol	7 (7)
**Total (at least one deviation)**	**15 (15.3)**

Data presented as no. (%).

Table S5. Secondary exploratory clinical outcome.

	Restrictive fluid group (n=46)	Usual care (n=49)	Median difference (95%HLCI)	*p*
Total volume of non-resuscitation fluids within 3 days of inclusion, ml§	3633 (2261-6414)	7334 (4039-9777)	-3316 (-4833--1954)	<0.001

Total volume of non-resuscitation fluids within 5 days of inclusion, ml[§]	4067 (2261-8985)	7977 (5088-13335)	-3191 (-5199--1166)	0.006
Total volume of resuscitation fluids within 3 days of inclusion, ml[§]	1354 (488-3265)	1578 (538-2722)	0 (-750-627)	0.77
Total volume of resuscitation fluids within 5 days of inclusion, ml[§]	1550 (613-3853)	1800 (538-3450)	37 (-742-680)	0.96
Cumulative fluid balance on day 3 from inclusion, ml[§]	249 (-1440-1985)	2317 (-588- 4242)	-2109 (-3480--831)	<0.001
Cumulative fluid balance on day 5 from inclusion, ml[§]	405 (-1173-2394)	2345 (-362- 4073)	-1812 (-3140--502)	0.002
Any acute kidney injury according to KDIGO within 90 d	39 (85)	38 (78)	-	0.37
Days alive and free of RRT within 90 d[¤]	90 (5-90)	90 (10-90)	0 (0-0)	0.75
Days alive with full enteral nutrition within 90 d*	79 (0-87)	77 (1-84)	0 (-3-4)	0.55
Highest dose norepinephrine first 5 days, mcg/kg/min	0.39 (0.21-0.51)	0.38 (0.2-0.5)	0.03 (-0.06-0.12)	0.37
Highest lactate first 5 days, mmol/l	3.8 (2.7-6.4)	4.3 (2.6-5.9)	-0.1 (-1-0.9)	0.93
Highest cardiovascular SOFA-score first 5 days	4 (4-4)	4 (4-4)	0 (0-0)	0.38
Number of days with diuretics, first 5 days	1 (0-2)	1 (0-2)	0 (0-0)	0.91
GOSE at 6 months¶	6 (4-8)	5 (4-7)	1 (-1-1)	0.74

Data are presented as the median (interquartile range) or number (%), as appropriate.
KDIGO – Kidney Disease Improving Global Outcome, RRT – renal replacement therapy, SOFA – Sequential Organ Failure Assessment, GOSE – Glasgow Outcome Scale Extended.
[§]Number of patients: 44 (restrictive fluid group) and 48 (usual care).
[¤]Number of patients: 45 (restrictive fluid group) and 48 (usual care).
*Defined as no need for intravenous nutrition or fluids.
[¶]Number of patients: 22 (restrictive fluid group) and 21 (usual care).
Differences in count outcomes are presented as median differences with 95% Hodge Lehmann (HL) confidence intervals (CI). All available data was used for all fluid calculations, i.e. fluid data includes data from patients staying less than three days.

Table S6. Complications and harms in the ICU up to 90 days after inclusion

	Restrictive fluid group (n=46)	Usual care (n=49)	*p*
≥ 1 complication in the ICU	39 (85)	38 (78)	0.37
- Ischemic complications	2 (4)	3 (6)	>0.99

▪ Limb	0	1 (2)	>0.99
▪ Cerebral	0	0	>0.99
▪ Heart	2 (4)	1 (2)	0.61
▪ Intestinal	0	1 (2)	>0.99
Hypoglycemia	10 (22)	4 (8)	0.08
Hypernatremia	1 (2)	0	0.48
Hyperchloremic acidosis	1 (2)	1 (2)	>0.99
Metabolic alkalosis	1 (2)	0	0.48
SUSAC	0	0	>0.99

Data presented as no. (%). SUSAC – Suspected unsuspected serious adverse complication. AKI is also considered an ischemic complication and is already presented in Table S5 and is therefore not displayed here, see table S5 for results. The Fisher's exact test was used for the statistical analysis.

Table S7. Post hoc-analyses of exploratory outcomes.

	**Restrictive fluid group** *n = 46*	**Usual care** *n = 49*	**Mixed effects linear regression** *estimate (95%CI)*	*p*	**Mixed effects logistic regression** *RR (95%CI)*	*p*
Δ Lactate, mmol/L<br>- missing*	-0.02 (-0.99;0.95)<br>5 (10.9%)	-0.38 (-1.16;0.39)<br>3 (6.1%)	-0.31 (-1.48;0.84)	0.60		
ΔCardiovascular SOFA<br>- missing*	-0.21 (-0.44;0.03)<br>7 (15.2%)	-0.11 (-0.35;0.14)<br>2 (4.1%)	0.10 (-0.24;0.44)	0.57		
Any diuretics within five days	29 (63.0%)	30 (61.2%)			0.97 (0.71;1.33)	0.86
RRT within 90 days<br>- missing¤	13 (28%)<br>1 (2.2%)	11 (22%)			0.76 (0.38;1.53)	0.44

Data presented as mean (95% CI) and no (%) as appropriate. Δ: Change in mentioned variable from baseline to maximum value within five days (D0-5). SOFA: sequential organ failure assessment, RRT: Renal replacement therapy.
*Patients admitted to the ICU for just one day will have only one daily value registered. The delta (Δ) value requires at least two registrations.
¤One patient was transferred to a non-study ICU.

Table S8. Acute kidney injury stage within 90 days

	Restrictive fluid group (n=45)	Usual care (n=48)	*p*
- AKI stage I	17 (37%)	15 (31%)	0.51
- AKI stage II	14 (30%)	11 (22%)	0.38
- AKI stage III	8 (17%)	12 (25%)	0.40

AKI: Acute kidney injury, according to KDIGO (Kidney Disease Improving Global Outcome). One patient in the restrictive fluid group and one in usual care was relocated to a non-study ICU. The χ^2-test was used for the statistical analysis.

Table S9. Participating hospitals and number of randomized patients.

Hospital	No.randomized
Kristianstad Hospital	32
Helsingborg Hospital	23
Skåne University Hospital, Malmö	18
Skåne University Hospital, Lund	12
Östersund Hospital	8
Halmstad Hospital	5

FIGURES

Figure S1. Daily volume of non-resuscitation fluids.

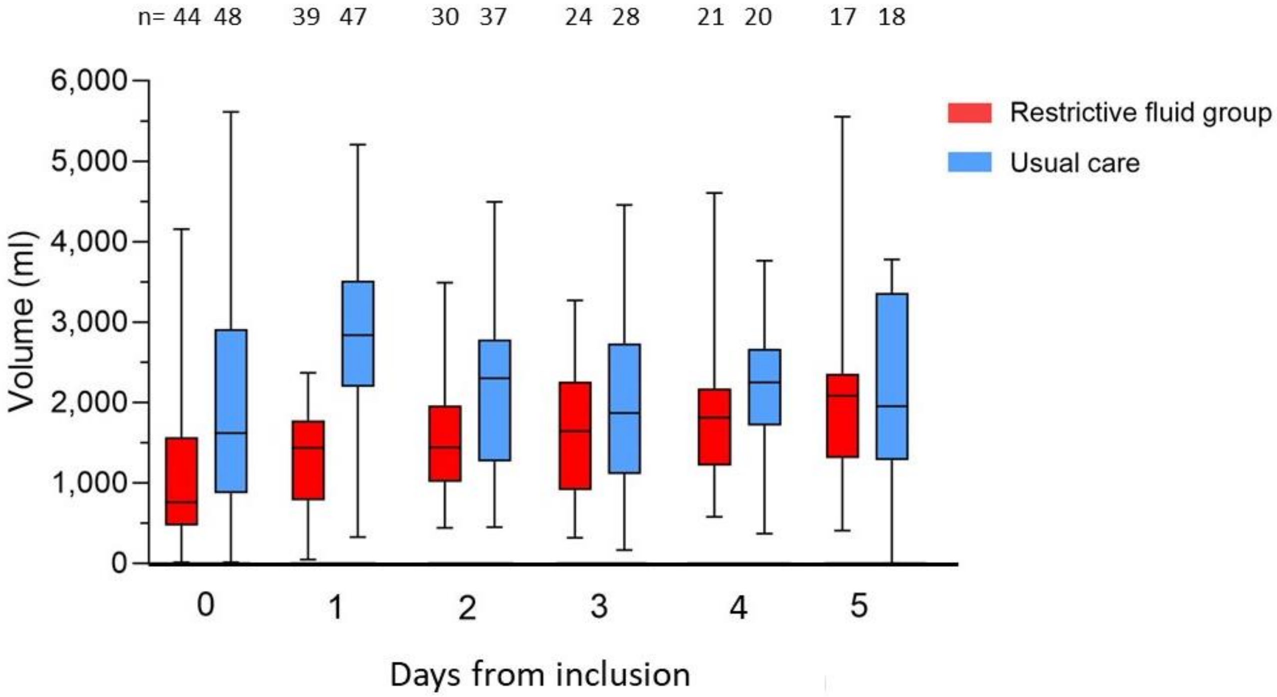

Volumes are presented as median, IQR and range. N indicates number of patients.

Figure S2. Daily volume of resuscitation fluids.

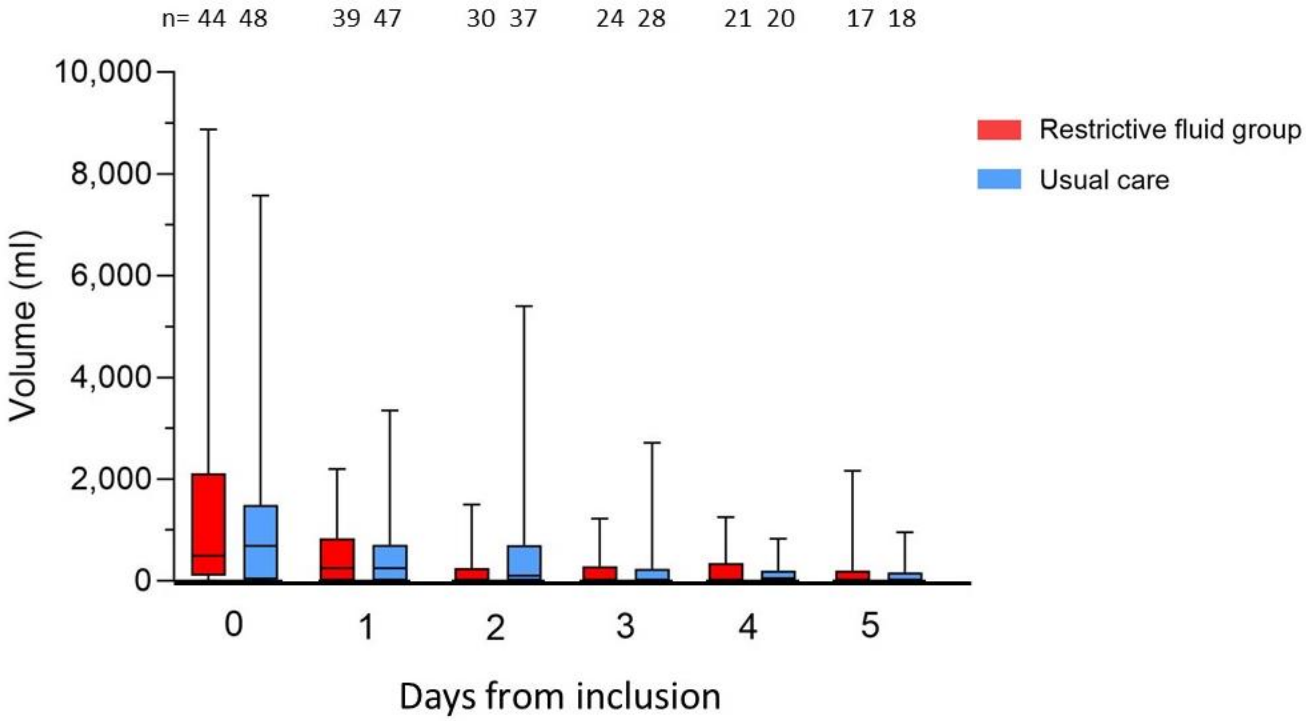

Volumes are presented as median, IQR and range. N indicates number of patients.

Figure S3. Cumulative fluid balance.

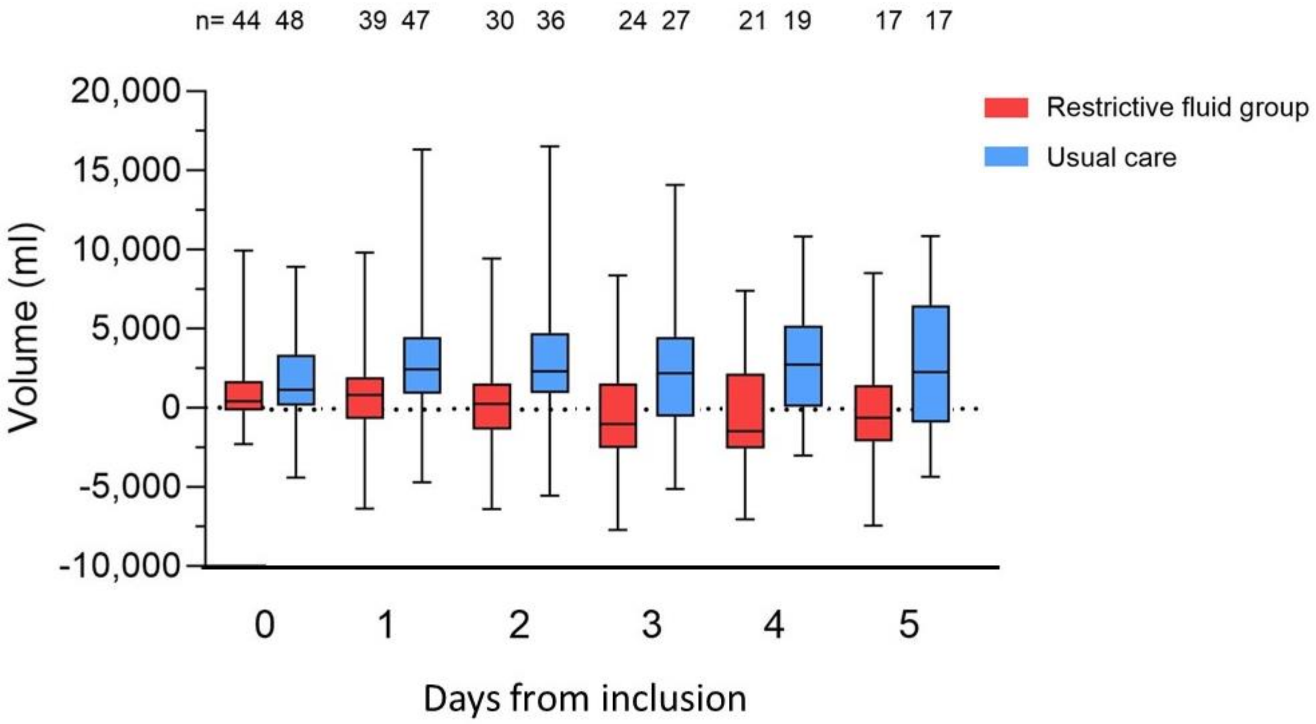

Volumes are presented as median, IQR and range. N indicates number of patients.

Figure S4. Cumulative total fluid administration.

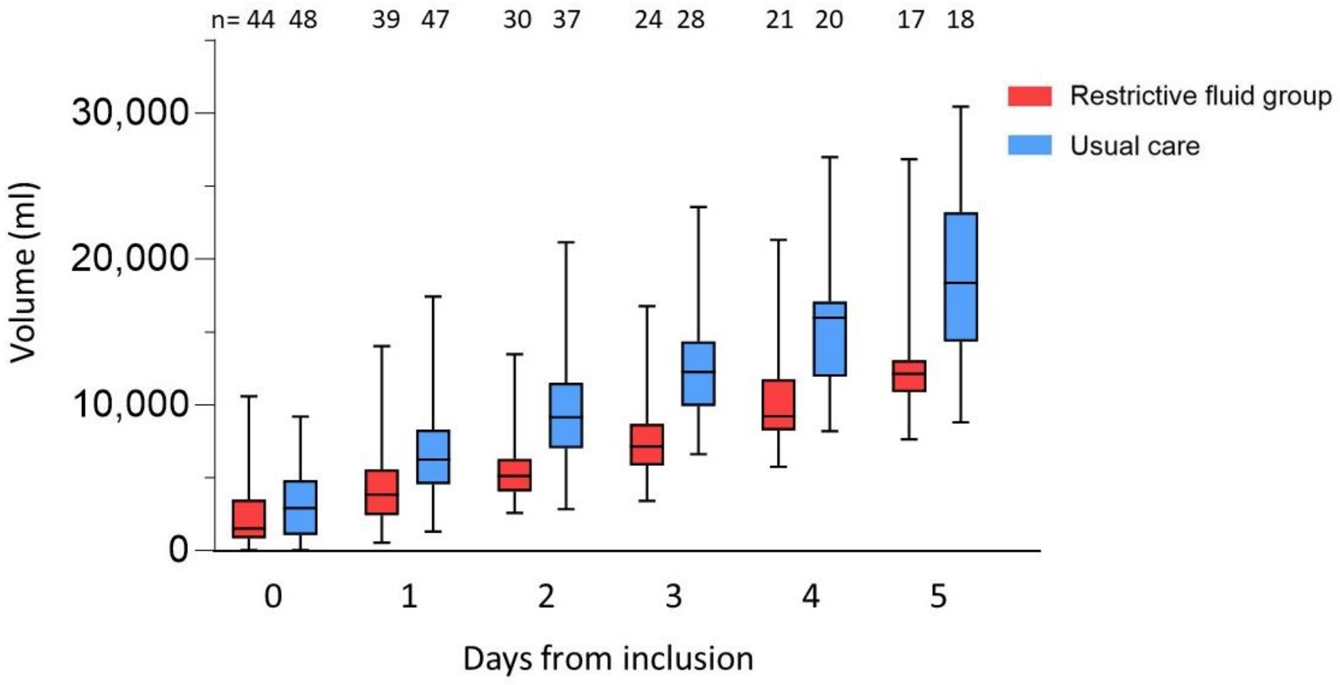

Volumes are presented as median, IQR and range. N indicates number of patients.

Figure S5. Survival 90 days from inclusion.

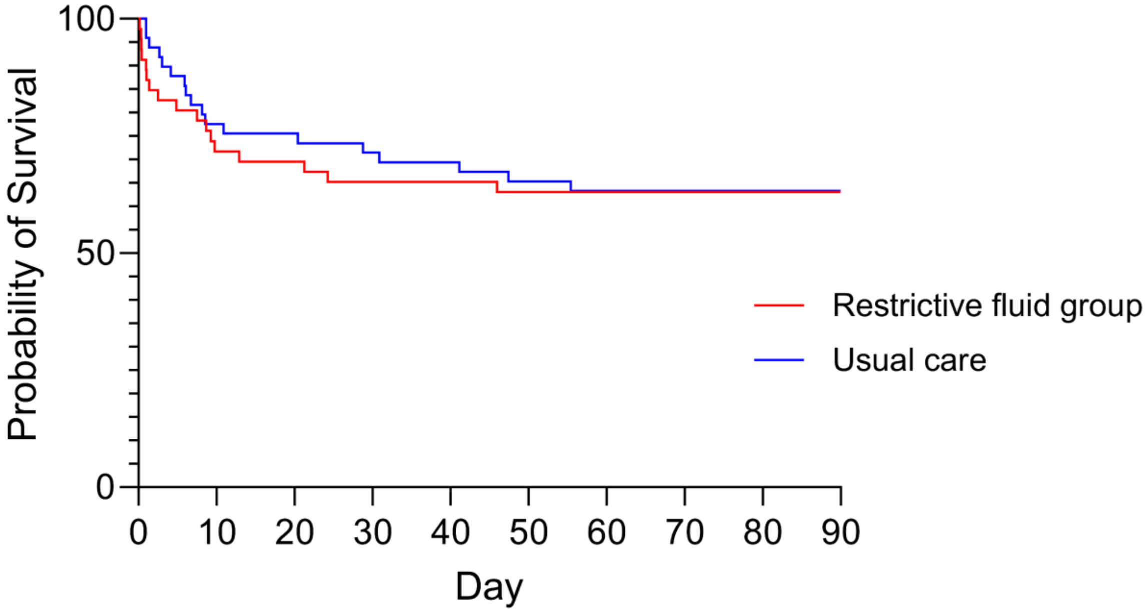

Kaplan-Meier plot displaying probability of survival over time in the two treatment arms.

APPENDIX A

Dilutions of medications in the intervention group.

MEDICATIONS in the intervention group. The concentrated solutions should only be used once the patient has a central line. To avoid waste of drug, apply protocol when it's time to change syringe. Glucose may also be supplied as a vehicle for medication rather than a separate infusion of 20% glucose. More concentrated solutions than those described below are allowed if already in use at trial site. Drugs not included in the table below should be used in the most concentrated dilution already in use at trial site.

**Drug**	**Conc. In stem solution**	**Suggested dilution in intervention group**	**Reference**	**Comments**
**Suggested dilutions may only be used if patient has a central line**<br>**Vasoactive drugs**				
**Adrenaline**	1 mg/ml	Start at 80 µg/ml and change to 160 µg/ml if infusion rate >10 ml/h	SPC, IM, Micromedex, Halmstad, UKCPA	**IM;** IV inf 40 – 320 µg/ml, diluted in G. **Halmstad**; 80 µg/ml in NaCl. **UKCPA**; Up to 500ug/ml has been used
**Amiodarone (Cordarone, Amiodaron Hameln)**	50 mg/ml	Dilute to 15mg/ml (20 ml G per 300mg amiodarone)<br><br>Note!<br>Dilute according to local guidelines in cardiac arrest,	SPC, ePED, UKCPA	**SPC**; Dilute in 5% G. **ePed;** 15mg/ml as infusion. **UKCPA**; Many centres infuse daily dose (up to 900mg) in a total volume of 48-50ml
**Dobutamine Hameln**	12,5 mg/ml	10 mg/ml	SPC, IM, UKCPA	**IM**; Fluid restr. Adult 2 amps (2x20 ml) + 10 ml NaCl/G give 10 mg/ml.

Drug	Conc. In stem solution	Suggested dilution in intervention group	Reference	Comments
**Isoprenaline**	0,2 mg/ml	use according to local protocol	Micromedex, Gahart´s, UKCPA	**Halmstad**; 10 ml 0,2 mg/ml in 40 ml G, gives 40 µg/ml. **Micromedex och Gahart´s**; recommend 20 ug/ml for iv bolus and 2 to 4 ug/ml for infusion.
**Milrinone**	1 mg/ml	use according to local protocol	SPC	**SPC** dilute to 200 ug/ml using G/NaCl.
**Nitroglycerin (Abcur och BioPhausia)**	1 mg/ml	use according to local protocol	SPC, IM	**SPC**; May be given undiluted using a pump. Can be diluted in G/NaCl. **IM**; 1 mg/ml may be given undiluted
**Noradrenaline (Abcur, Pfizer)**	1 mg/ml	Start at 80 µg/ml and change to 160 µg/ml if infusion rate >10 ml/h	SPC, IM, Micromedex, Stabilis	**SPC**; Noradrenaline 1 mg/ml should be diluted with G/NaCl before use. **IM**; 160 µg/ml. **Micromedex**; G may protect against oxidation. **Stabilis**; 0.5 mg/ml Norepinephrine bitartrate is stabile in G for 48 h at 20-25 °C.
**Levosimendan**	2.5 mg/ml	0.05 mg/ml (10 ml levosimendan 2,5 mg/ml in 500 ml G	SPC, IM	
**Phenylefrine (Abcur och Unimedic)**	0.1 mg/ml	use according to local protocol	Micromedex, IM	**Micromedex**; for iv bolus use 100 µg/ml and 20 µg/ml for inf.
**Vasopressin/ Argipressin (Empressin)**	20 IE/ml	0.4 E/ml	IM, UKCPA	**IM;** 1 amp. (1 ml, 20 units in 50 ml med G,

Drug	Conc. In stem solution	Suggested dilution in intervention group	Reference	Comments
				will give conc 0.4 units/ml. **Gahart´s;** 1 E/ml
**Antibiotiotics**				
**Acyklovir**	25 mg/ml	5 mg/ml Dilute 10 ml 25 mg/ml with 40 ml of NaCl/G	SPC, UKCPA	**UKCPA;** 25mg/ml over 1 hour by controlled rate infusion. If diluted 5mg/ml infused over at least 1 hour**.**
**Ampicillin**		1 g in 10 ml of sterile water<br><br>2 g in 20 ml of sterile water	SPC, Micromedex	**SPC;** For iv inj. 10 and 20 ml for 1 and 2 g, respectively. **Micromedex;** 1 and 2 g may be diluted in 7.4 and 14.8 ml sterile water, respectively and given in 10-15 min to minimize risk of seiziures. **SPC Meda/Mylan**. Give slowly (minimum 3-4 minutes).
**Anidulafungin**		100 mg in 30 ml of sterile water and add to 100 ml G/NaCl.	SPC, Stabilis, Micromedex, Gahart´s	Infusion rate 1,4 ml/min resulting a total infusion time of 90 min.
**Bensylpenicillin**		1 g in 10 ml of sterile water<br><br>3 g in 20 ml of sterile water	SPC, IM	**SPC;** dissolve 1 g in 10 ml of sterile water and 3 g in 20-40 ml of sterile water. **IM;** 600 mg in 4-10 ml. Inject slowly (> 3-5 minutes)
**Caspofungin**	50 mg	Carefully dissolve 50 mg in 10,5 ml of sterile water. Add to 100 ml NaCl.	Micromedex, SPC, IM	Give drug during at least 60 min!

**Drug**	**Conc. In stem solution**	**Suggested dilution in intervention group**	**Reference**	**Comments**
	70 mg	Carefully dissolve 70 mg l in 10,5 ml of sterile water. Add to 140 ml NaCl		
**Cefotaxim**		1 g in 4 ml sterile water<br><br>2 g in 10 ml sterile water	SPC	**SPC**; Note that rapid injection in central line has been reported to cause life threatening arrythmia in rare cases.
**Ceftazidim**		1 g in 10 ml of sterile water<br>2 g in 10 ml of sterile water	SPC, IM, Micromedex	
**Ceftriaxon**		1 g in 10 ml of sterile water<br>2 g in 20 ml of sterile water	SPC, Stabilis, IM. Micromedex	**SPC**, Use NaCl or G for 2 g. **Stabilis;** 100 mg/ml in water is ok. **Micromedex;** 2 g in 10 ml. **IM;** Infusion if dose ≥ 2 g.
**Cefuroxim**		750 mg in 6 ml of sterile water<br><br>1,5 g in 15 ml of sterile water	SPC, IM	
**Clindamycin**	150 mg/ml	600 mg in 50 ml G/NaCl (gives 11 mg/ml)	SPC, IM	**IM;** Final concentration max 18 mg/ml. **SPC, IM;** shortest infusion time is 600 mg in 20 min.
**Cloxacillin**		1 g in 20 ml of sterile water	SPC, Stabilis	**Stabilis** conc up to 250 mg/ml are ok.

Drug	Conc. In stem solution	Suggested dilution in intervention group	Reference	Comments
		2 g in 40 ml of sterile water		
**Doxycyklin**	20 mg/ml	100 mg in 100 ml G/NaCl<br><br>200 mg in 200 ml G/NaCl	SPC	
**Erytromycin**		1 g in 20 ml sterile water and add 80 NaCl.	IM, ePed, SPC,	**IM**; Final concentration should not be greater than 10 mg/ml. **ePed;** Give dose in > 1h to mimimize risk of arrythmias.
**Gentamycin**	40 mg/ml	May be given undiluted as bolus.<br><br>Repeated doses either diluted or as boluses over 3-5 minutes depending on dosing regimen.	SPC, UKCPA	**SPC**; If administered twice daily gentamycin may be given undiluted in 3-5 minutes. **UKCPA**; For large doses most centers dilute with 50 ml G/NaCl.
**Imipenem/Cilastatin**		500/500 mg in 10 ml NaCl and add to 90 ml NaCl/G. Maximum concentration of imipenem 5 mg/ml	IM, SPC, UKCPA	**SPC;** doses ≤ 500 mg/500 mg should be given over 20 to 30 minutes and doses >500 mg/500 mg should be given over 40 to 60 minutes.
**Meropenem**		For bolus dilute in sterile water to a final	IM, SPC, UKCPA	**IM;** 0.5–- 1 g doses in 5 min. 2 g doses in 15-30 min. **SPC**. Meropenem diluted to 20 mg/ml in

Drug	Conc. In stem solution	Suggested dilution in intervention group	Reference	Comments
		concentration of 100 mg/ml<br><br>For infusion dilute to 20 mg/ml with NaCl		NaCl stable for 3 h in room temperature.
**Metronidazol**	5 mg/ml	Undiluted		
**Piperacillin/Tazobactam**		2/0,25 g in 10 ml of sterile water/NaCl<br><br>4/0,5 g in 20 ml of sterile water/NaCl<br><br>For infusions dilute further with G/NaCl to 50 ml	SPC, IM,	
**Tobramycine**	40 mg/ml	Use undiluted	IM, SPC	
**Tobramycine**	80 mg/ml	80 mg/ml dilute with 50 ml G/NaCl	SPC	**SPC;** shorter infusion time than 20 minutes will increase risk for toxic side-effects and is not recommended.
**Trimetoprim/Sulfametoxazol**	16+80 mg/ml (5 ml/amps)	2 amps. in 150 ml G.<br>Observe carefully for precipitates.<br><br>4 amps. in 300 ml G	SPC, IM	**SPC;** Stable for 2 h! **IM;** possible to give undiluted stock solution in 60-90 min (off label).
**Vancomycin**		500 mg in 10 ml sterile water. Add to 40 ml NaCl/G	IM, UKCPA	**IM;** In exceptional circumstances 20 mg/ml may be given via a central line. **UKCPA;** 10

Drug	Conc. In stem solution	Suggested dilution in intervention group	Reference	Comments
		to give a conc. of 10 mg/ml<br><br>1 g in 20 ml of sterile water. Add to 80 ml NaCl/G to give a conc. of 10 mg/ml		mg/ml is a commonly used dilution. 20 mg/ml has been used in some centers. **IM;** give in 1 h. **Regional dilution routine;** Give a dose of 500 mg in 60 min and 1 g in a 100 min.
**Vorikonazole**	200 mg	200 mg in 19 ml of sterile water to a conc. of 10 mg/ml. For doses 50-500 mg: add to 100 ml G/NaCl. For doses > 500 mg: add to 250 ml G/NaCl.	IM, SPC	Final concentration should be 0,5-5,0 mg/ml. Max infusion rate is 3 mg/kg/h.
**Fluconazol**	2 mg/ml	Use undiluted	SPC	Infusion rate 10 ml/min or lower.
**Other drugs**				
**Clonidine**	150 µg/ml (1 ml ampull)	30 µg/ml	SPC, IM, UKCPA	**UKCPA;** 6-50 micrograms/ml infusion. Diluent: Sodium chloride 0.9% or glucose 5%.
**Dexmedetomidine**	100 ug/ml	8 ug/ml	SPC	
**Sodium glycerophosphate (Glycophos)**	1 mmol/ml	0.5 mmol/ml<br>20 ml sodium glycerophosphate 1mmol/ml in 20 ml NaCl.	ePED	**ePED**; Administer in no less than 8 h.
**Insulin (Humulin Regular, Actrapid)**	Insulin, humant	1 E /ml	Stabilis	**Stabilis;** Dilute in NaCl

Drug	Conc. In stem solution	Suggested dilution in intervention group	Reference	Comments
	(Humulin Regular)			
**Levetiracetam**	100 mg/ml (5 ml flaska)	250 - 1500 mg in 100 ml NaCl/G, administer in no less than 15 min.	SPC, IM, Micromedex, Gahart´s	**Micromedex;** Do not exceed a final max cons of 15 mg/ml. Can be given as iv bolus, 3-5 min and cont infusion 200-400 mg/h.
**Magnesium sulphate (Addex-Mg**)	1 mmol/ml	0.5 mmol/ml, (20 ml in 20 ml NaCl, giving a conc. 0,5 mmol/ml). Give in no less than 10 min.	IM, Gahart´s, VGR guideline, UKCPA	**Gahart´s;** D5W and NS are the most common diluents. **UKCPA**; suggested dilutions 1-2mmol/ml
**Potassiumhydroxide/ Potassium phosphate (Addex-Kalium)**	2 mmol/ml	1-2 mmol/ml dilute in NaCl if needed	SPC	**SPC;** Give at most 20 mmol potassium/h.
**Potassium Chloride**	2 mmol/ml	1-2 mmol/ml, in NaCl dilute if needed	SPC	**SPC;** Give at most 20 mmol potassium/h.
**Propofol (Propofol-Lipuro)**	10 or 20 mg/ml	20 mg/ml for infusion. According to local routine for intubation	SPC	

NaCl = sodium chloride 9 mg/ml = NS, **G=**Glukose 50 mg/ml=Dextrose 5%=D5W

ePed = experience and evidence-based database for pediatric medicnes, https://eped.se, **Gahart´s** = Gahart´s 2021 intravenous medication via https://www.clinicalkey.com. **Halmstad** = vårdriktlinje "Inotropa läkemedel och vasopressorer HSH" published in 200913, **IM** = UCL Hospitals Injectable Medicines Administration Guide: Pharmacy Department, 3rd Edition, University College London Hospitals, ISBN: 978-1-405-19192-0, **Micromedex** = https://www.micromedexsolutions.com, **Regional dilution routine Region Skåne, Sweden** =

www.lakemedelshantering.se, **SPC** = Summary of product characteristics, **Stabilis** = https://www.stabilis.org, **UKCPA** = United Kingdom Clinical Pharmacy Association: Minimum infusion volumes for fluid restricted critically ill patients. 4th edition Dec 2012

APPENDIX B

Treatment algorithm for non-resuscitation fluids in the intervention group.

Enteral nutrition: 2 kcal/ml, start according to local protocol.
Parenteral nutrition: according to local protocol.
Intravenous fluid and enteral water: according to need, to correct electrolyte disturbances, according to local protocol.
Medications and electrolytes: according to separate medication protocol (Appendix A).
Maintenance fluid: see flowchart below.

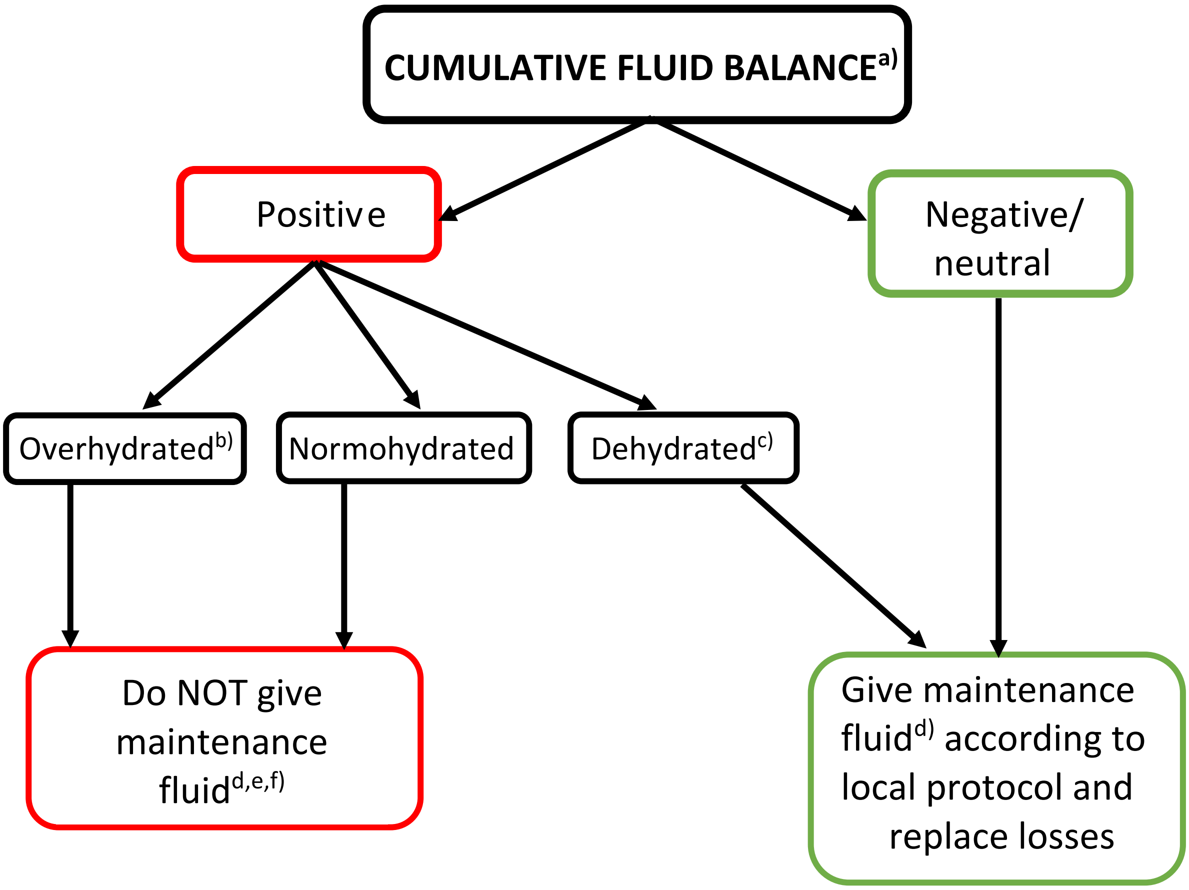

[a] Measured ins and outs:
In: Nutrition, maintenance fluid, medications and electrolytes, crystalloids given to correct hemodynamic instability, blood transfusions and colloids.
Out: Diuresis, fluid removal from dialysis, fluid drainage, vomiting/GI-tubing, bleeding, and fluid from fecal systems such as Flexi-Seal.
[b] Overhydrated (increased total body water in relation to baseline), indicated by weight over baseline/admission weight and/or peripheral/radiological oedema.
[c] Dehydrated (decreased total body water in relation to baseline), indicated by weight below baseline/admission weight, decreased skin turgor, dry mucus membranes. Adjust baseline weight to estimated weight loss during ICU stay.
[d] Maintenance fluid is defined as intravenous fluid (crystalloids not administered to correct hemodynamic instability, and/or glucose solutions) or enteral water prescribed to ensure that total volume of fluid administration covers the daily fluid requirements (approximately 1 ml/kg/h).
[e] Starting at 72 hours after randomization, glucose solutions may be prescribed at a maximum dose of 1g/kg/day, if enteral nutrition is not tolerated. Glucose solutions, at this dose or lower, may be started earlier than day 4 in patients with insulin dependent diabetes, if enteral nutrition is not tolerated and if local protocol demands it. The minimum concentration must be 20% unless the patient is dehydrated.
[f] Diuretics can be prescribed to reach the desired fluid balance.

REFERENCES

1. Kellum JA, Lameire N, KDIGO AKI Guideline Work Group. Diagnosis, evaluation, and management of acute kidney injury: a KDIGO summary (Part 1). Crit Care 2013;17:204.
2. Finfer S, Liu B, Taylor C, et al. Resuscitation fluid use in critically ill adults: an international cross-sectional study in 391 intensive care units. Crit Care 2010; 14:R185.
3. Inker LA, Eneanya ND, Coresh J, et al (2021) New creatinine- and cystatin C-based equationsto estimate GFR without race. New Engl J Med 385 (19):1737-1749.
4. Mellhammar L, Elén S, Ehrhard S, et al (2022) New, Useful Criteria for Assessing the Evidence of Infection in Sepsis Research. Crit Care Explor. 4:e0697. doi:10.1097/CCE.0000000000000697
5. Rockwood K, Song X, MacKnight C, Bergman H, Hogan DB, McDowell I et al (2005). A global clinical measure of fitness and frailty in elderly people. CMAJ Can Med Assoc J J Assoc Medicale Can. 173:489–95.
6. Lindén-Søndersø, A, Jungner, M, Spångfors, M et al. Survey of non-resuscitation fluids administered during septic shock: a multicenter prospective observational study. Ann. Intensive Care 2019; doi:10.1186/s13613-019-0607-7
